# Data-Driven Astrochemistry: One Step Further within the Origin of Life Puzzle

**DOI:** 10.3390/life8020018

**Published:** 2018-06-01

**Authors:** Alexander Ruf, Louis L. S. d’Hendecourt, Philippe Schmitt-Kopplin

**Affiliations:** 1 Analytical BioGeoChemistry, Helmholtz Zentrum München, Ingolstaedter Landstr. 1, 85764 Neuherberg, Germany; 2 Analytical Food Chemistry, Technische Universität München, Maximus-von-Imhof-Forum 2, 85354 Freising, Germany; 3 Université Aix-Marseille, Laboratoire de Physique des Interactions Ioniques et Moléculaires (PIIM), UMR CNRS 7345, 13397 Marseille, France; ldh@ias.u-psud.fr

**Keywords:** astrochemistry, meteoritics, ultrahigh-resolving analytical chemistry, data analysis, origin of life

## Abstract

Astrochemistry, meteoritics and chemical analytics represent a manifold scientific field, including various disciplines. In this review, clarifications on astrochemistry, comet chemistry, laboratory astrophysics and meteoritic research with respect to organic and metalorganic chemistry will be given. The seemingly large number of observed astrochemical molecules necessarily requires explanations on molecular complexity and chemical evolution, which will be discussed. Special emphasis should be placed on data-driven analytical methods including ultrahigh-resolving instruments and their interplay with quantum chemical computations. These methods enable remarkable insights into the complex chemical spaces that exist in meteorites and maximize the level of information on the huge astrochemical molecular diversity. In addition, they allow one to study even yet undescribed chemistry as the one involving organomagnesium compounds in meteorites. Both targeted and non-targeted analytical strategies will be explained and may touch upon epistemological problems. In addition, implications of (metal)organic matter toward prebiotic chemistry leading to the emergence of life will be discussed. The precise description of astrochemical organic and metalorganic matter as seeds for life and their interactions within various astrophysical environments may appear essential to further study questions regarding the emergence of life on a most fundamental level that is within the molecular world and its self-organization properties.

## 1. Astrochemistry and Meteoritics

Astrochemistry and meteoritics study chemical processes within different astrophysical environments, including the interstellar medium (ISM) or circumstellar and circumplanetary regions [1]. The evolution, transport and transformation of molecules are monitored, from diffuse ISM and dense molecular clouds to their accretion/incorporation into minor bodies of the solar system such as asteroids and comets. These solar system objects represent the parent bodies of meteorites that are found even today on the Earth’s surface.

This scientific field combines many aspects from different disciplines, which may benefit from each other. Roughly speaking, it includes “everything”, as considering the need for tracking the origin and chemical evolution of our Galaxy up to possible implications on the origin of life, not only on Earth, but also on the other many worlds that are being discovered nowadays. Astrobiology focuses on questions on the origin of life or questioning the habitability concept of planets other than our own. Motives within different scientific techniques from astronomy, astrophysics and chemistry (physical/theoretical/analytical chemistry) are combined to gain molecular information from space environments. Additionally, biochemists and biologists may complement these multiple areas of research to work in particular on astrobiological problems such as the origin of life or the habitability of other planets. Even interests for philosophers and social sciences are set in these topics, which influence many social and cultural aspects of our civilization.

### 1.1. Interstellar Medium and Circumstellar Environments

The detection of molecules within the ISM and circumstellar environments is achieved mainly via radio astronomy. For a long time, scientists suspected that the interstellar medium’s environment would be too harsh for organic species to survive and that only a few simple molecules could be formed under such extreme conditions. However, since the 1970s, millimeter and submillimeter observations have detected to date ≈200 interstellar and circumstellar molecules, including hydrocarbons, alcohols, carboxylic acids, aldehydes, ketones, amines, ethers and other organic molecules (Table 1) [2]. In addition, 62 extragalactic molecules were detected [2]. Modern molecular spectroscopy studies led to the detection of this seemingly large number of molecules in astrophysical environments. Most of them have been discovered via their rotational signatures from radio to far-infrared frequencies. In addition, some have also been observed in the visible and near-infrared domains.

Technically, infrared ground-based, airborne and space-based spectroscopic observations have found evidence for complex organic molecules (COMs) with both aromatic and aliphatic structures in space. As a striking example, the Atacama Large Millimeter/submillimeter Array (ALMA) ground-based observatory from European Southern Observatory (ESO) should be mentioned here [3]. For example, powerful scientific results such as studies on cosmic dust [4], comae of comets [5] or protoplanetary disks surrounding stars [6] were obtained. A second breakthrough example in radio astronomy is the Herschel Space Observatory, a space observatory of the European Space Agency (ESA), collaborating with the National Aeronautics and Space Administration (NASA) [7]. Striking results on planet formation could be reported with the help of Herschel observations [8].

### 1.2. Molecular Complexity

The detected diverse set of molecules in space is statistically connected with the term molecular complexity [1,9]. As molecular complexity represents properties for a single molecular type, chemical diversity is proportional to the number of different compounds in a chemical system of interest. Both terms, molecular diversity and molecular complexity, need to be treated separately and should not be confused with each other. Complexity is an important concept in many scientific disciplines. Nevertheless, it is challenging to define and to quantify to date [10]. A complex system or a complex molecule can evolve in multiple ways, often following non-linear processes. Complex systems consist of a large number of degrees of freedom as compared to a less complex one [11].

In terms of molecular complexity, attempts have been made with the help of information theory and graph theory [12,13,14,15,16]. These methods enable coarse measures of molecular complexity and allow for its quantification [17]. Qualitatively, molecular complexity scales with molecular size, heteroelements and functional-group contents within a given molecule, cyclic connectivity/branching ratios, stereocenter contents and chemical reactivity [18]. One example is the isomerization of bromobutane, which expresses the relation of complexity with the degree of freedom in a certain molecule [19].

In the astrochemical context, molecular complexity is probed following two methodologies, namely via a bottom-up approach or a top-down one.

The bottom-up approach, which is common in astrochemistry, considers the precise description of fundamental building blocks and studies the ongoing formation of increasingly more complex species, based on the described building blocks and their behavior. In terms of astrochemistry, characteristics of small diatomic species and their transformation into gradually more complex polyatomic molecules are proposed. Probing the ISM can be related to gas-phase physical chemistry, which mostly consists of gas phase reactions or grain-surface reactions [1]. Molecular complexity is usually studied by chemical reaction networks for molecules bearing less than 10 atoms. Reaction networks elucidate basic elemental/molecular reactions [1]. This bottom-up approach gives ideas about fundamental reaction pathways within the formation of complex organic molecules (COMs).

The top-down approach targets highly complex organic molecules, e.g., in meteoritic samples. Here, the formation of COMs is tentatively deciphered via retrosynthesis. Potential precursors of these COMs are analyzed to gain ideas about possible formation pathways of prior detected COMs. Herein, a significantly higher number of diverse and complex organic molecules (much more than 10,000 molecules with molecular weights up to 750 atomic mass units (amu)) can be observed. These compounds, as detected by comprehensive meteoritic analyses, can be further studied within a chemical network to get insights into possible precursors for complex organic species [20,21].

Both analytical strategies are complementary and tentatively converge in information and try to describe problems on astrochemical molecular complexity as comprehensively as possible.

### 1.3. Chemical Evolution

When speaking about the diversity and complexity of organic molecules, one simultaneously asks for the origin and temporal evolution of these complex organic species [22]. The term chemical evolution is ambiguously discussed nowadays [23]. Originally, Calvin introduced this term to describe “the conversion of simple organic or inorganic molecules to assembled, complex, partly polymeric chemical compounds, which eventually became capable of reproduction including mutation and metabolism” [23,24]. Nevertheless, the term is also interpreted and used to denote processes of astrophysical synthesis of small molecules [25].

In this review, chemical evolution is used to combine a general concept of the formation and evolution of chemical molecules, starting from astrochemical synthesis within interstellar environments toward their interactions/transformation within prebiotic chemical systems. Here, a prebiotic chemical system is expressed as a state that is irreversibly connected to a biotic/living state (e.g., via autocatalytic processes) [26] and is assumed to be a physical state far from thermodynamic equilibrium. The transformation of abiotic compounds toward those in biotic systems follows an increase in molecular complexity, essentially related to the organization of compounds and networks that select these compounds. As an illustration, polymeric biomolecules (e.g., ribonucleic acid, RNA) are more complex than astrochemical building blocks (e.g., methanol). While the complexity of molecules increases with chemical evolution, the complexity of a chemical system decreases and becomes less diverse.

One brief example is the chemical evolution of amino acids. There is a decrease in the number of amino acids from 92, as found in meteorites [27,28,29], toward 22 proteinogenic amino acids only within all biochemical systems on Earth [30]. In meteorites, only twelve proteinogenic amino acids (glycine, alanine, aspartic acid, glutamic acid, serine, threonine, proline, valine, leucine, isoleucine, phenylalanine and tyrosine) have been identified to date [29]. This finding makes those twelve amino acids potentially interesting for studying a minimal basis of biochemically-relevant molecules. Are the other proteinogenic amino acids formed within a later step in chemical evolution, maybe under prebiotic or biotic conditions?

Computational chemistry methods have been applied to address the question of what makes proteinogenic amino acids specific and why these are used in particular in biochemical systems [31,32]. The understanding of the evolutionary selection of the 22 particularly used proteinogenic amino acids in biotic systems on Earth has remained elusive. What are the driving forces to select only ≈31% of amino acids for biotic organized systems; what makes the 22 proteinogenic amino acids so special? Is this apparent chemical convergence in the number of amino acid compounds in observed biotic systems stochastic/random or truly deterministic, and is it universal to all living species on any planet or related to precise environmental conditions that prevailed a long time ago on Earth? Additionally, it has been shown that meteorites reveal also a much higher degree of molecular diversity for other chemical classes than that found in any organic matter of terrestrial origin [20,33].

More data on astrochemical organic diversity and its transition toward a less diverse set of organic molecules within biospheres may help to enable more precise descriptions of this molecular convergence within geological time scales. In turn, this might enable one to answer prior questions on the significance of determinism within chemical evolution, a question that is central in modern biology. This process might well be deterministic, but, at the same time, certainly depending on a huge number of parameters and contingencies that were linked to specific physical environments on different planets. These parameters will make life on any other planet most probably different from ours even if based on the same organic chemistry and physical principles (e.g., far from equilibrium chemistry). Specific different physical conditions will prevail on different planets, as already evidenced by the detection and characterization of many, very different exoplanets [34].

Discussing chemical evolution in general, it is necessary to address the stability of a molecule as generally as possible. A molecule’s life-time is highly dependent on the following parameters:

- astrophysical energy gradients and sources
- combinatorial effects of molecular synthesis, influenced by local elemental abundances
- intrinsic life-times of molecules (chemical stability)
- encapsulation within stabilizing molecular structures (early molecular preservation)
- molecular assembly potential (minimal chemical communication systems)
- molecular environments as compartmentalized systems (different energetic conditions)

**Figure 1:**
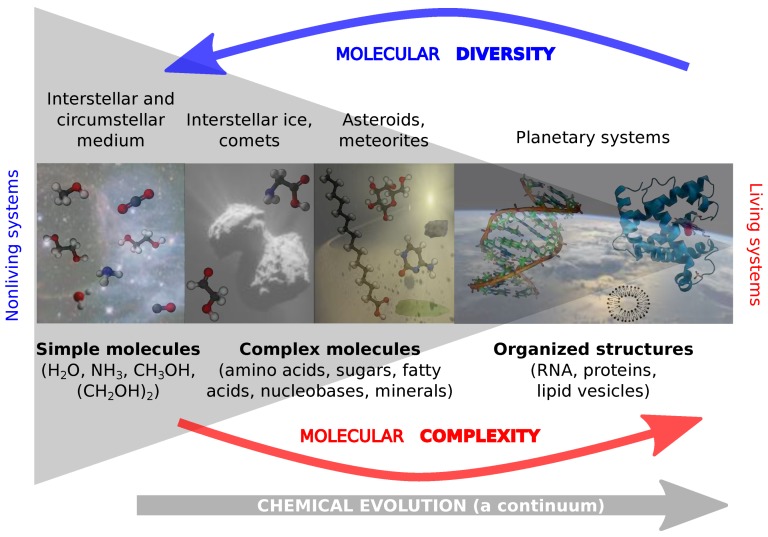
Sketching chemical evolution in terms of molecular diversity and molecular complexity. Molecular transformation within time and space is illustrated. Simple molecules within interstellar and circumstellar media evolve to highly-oriented, organized, complex macromolecules on planetary systems, enabling the potential of living systems.

Figure 1 sketches the chemical evolution in terms of molecular diversity and molecular complexity. Chemical evolution might be rather continuous and not a compartmentalized process. Starting within the interstellar and circumstellar media with simple precursor molecules (e.g., H${}_{2}$O, NH${}_{3}$ or CH${}_{3}$OH), which form more complex (mostly organic) molecules, chemical evolution and transformation toward rather complex biomolecules (e.g., RNA or proteins) is triggered by highly diverse, complex and multiparametric circumstances [35]. Chemical evolution and molecular complexification might go “hand in hand” with material accretion of astronomical/celestial bodies (e.g., formation and evolution of dust particles). This is indicated by sampling different objects within the timeline of chemical evolution (ISM, comets, meteorites, planets). In line with chemical evolution as going from astronomical media toward planetary systems, a decrease in molecular diversity can be noted [20,33]. In parallel, molecules become more complex and organized on planetary systems as compared to the interstellar medium. Organized molecules such as RNA or lipids (vesicles) are able to form biochemical structures, e.g., via hydrogen bonds.

### 1.4. Comet Chemistry

Comets represent astronomical objects that are “one step further” within chemical evolution with respect to interstellar or circumstellar media. Comets originate either from the Kuiper belt or from the Oort Cloud, as formed in the outer regions of the solar system. The Kuiper belt is a disc-shaped collection of icy debris at a distance of around 100 astronomical units (AU) just outside the orbit of Neptune. The Oort Cloud extends from 20,000–100,000 AU from the Sun. The cloud may contain a significant mass of material left over as a remnant of the formation of the solar system. A present-day comet represents a good probe to study the composition of the outer solar system and, more interestingly, the early solar nebula [36].

A milestone in probing in situ comet chemistry was the Rosetta mission, via a space probe built by ESA, along with its lander module Philae [37]. The comet 67P/Churyumov–Gerasimenko was visited. This mission was named “Rosetta”, launched on 1 March 2004. The mission ended on 30 September 2016. In the astrochemical context, special focus was on the search for organic molecules on 67P/Churyumov–Gerasimenko. Philae’s COSAC (Cometary Sampling and Composition experiment) instrument (a gas chromatograph, GC and a time-of-flight mass spectrometer, TOF-MS) was designed to identify organic compounds in the material from the nucleus of the comet. Second, ROSINA (Rosetta Spectrometer for Ion and Neutral Analysis) was used to study organic compounds within the comet’s atmosphere and ionosphere. ROSINA consists of two mass spectrometers, a double focusing magnetic mass spectrometer (DFMS) and a reflectron-type time-of-flight mass spectrometer (RTOF). Especially, the DFMS data are of high interest because of their high mass resolving power *R* (=3000), which allows for differentiating ${}^{12}$C${}^{16}$O from ${}^{14}$N${}_{2}$ or ${}^{13}$C from ${}^{12}$CH within a mass range of 1–150 amu (atomic mass units). Various organic molecules, including the smallest amino acid glycine [38] or high-molecular-weight organic compounds [39], were detected by the Rosetta mission.

### 1.5. Laboratory Astrophysics

Laboratory astrophysical studies are a contemporary and important tool to “solve puzzles” in the field of astrochemistry, as well as to complement observational radio astronomy, astrophysical modeling and state-of-the-art chemical analysis of meteoritic samples [40]. Technical detection problems within observational approaches can be overcome by simulating, e.g., interstellar ice analogs in the laboratory. This enables a better understanding of the formation mechanisms of complex organic molecules [41], predicting reactive intermediate species [42], overcoming spectroscopic detection challenges [43] or understanding celestial bodies’ evolution [44].

Going one step further in chemical evolution is performed via analyzing meteorites, possessing mineral components and even rocky materials. This brings meteoritic bodies closer toward planetesimal characteristics, in terms of geology. Next to comets, most asteroids (meteoritic parent bodies) represent astronomical objects that are thought to have survived from the very beginnings of the solar nebula some 4.5 billion years ago [36]. The analysis of meteorites sheds light onto astrochemical complexity, as will be further discussed within the next sections. We will see further benefits of laboratory chemical analysis.

## 2. Meteorites

Hot cores in giant molecular clouds collapse to form young stellar objects, the birth of stars via protostars’ formation. Newly-formed stellar objects include their own cloud or nebula around them. Analogously, the solar nebula was nothing more than an average collection of gas and dust that had achieved a critical mass. Molecular cloud collapses may have been triggered by shock waves, perhaps from supernovae, accelerating the rate of collapse so that above a critical mass, the collapse was inevitable. Shock waves are mainly believed to be collisionless plasma instabilities including extremely high energy particles, traveling through space [45,46]. Meteoroids are aggregated dust particles as derived from a giant molecular cloud. The early composition of the solar nebula was fundamentally responsible for the composition of the Sun and, with some further significant and large-scale processing, for the chemistry of the planets. In other words, the early composition of the solar nebula is directly connected to our contemporary “chemical household”, as possibly being the chemical basis of life.

Together with comets, asteroids (and thus, meteorites) may as well be called “fossils” or “children” of our solar system. Meteorites are thought to have survived since the birth of the solar nebula, some 4.5 billion years ago [36,47]. These stony samples profit from many aspects relative to other types of extraterrestrial materials. As meteorites are found on Earth, these types of samples can be analyzed in chemical laboratories using high-end analytical instrumentation. A high degree of analytical precision allows for sensitive probing of, e.g., the early solar system or planetary history information [48,49]. Many more powerful channels of information are provided by analyzing meteorites such as to profile astrochemical diversity and complexity.

First, we want to clarify the terminology on a meteor, meteoroid and meteorite [36].

Meteoroid: an object coming from a comet or asteroid orbiting the Sun.

Meteor: an object entering Earth’s atmosphere that burns up completely during its passage through the upper atmosphere; a ‘shooting star’.

Meteorite: an object that survives its entry through the Earth’s atmosphere and lands upon the Earth’s surface, is recovered and can thus be sampled.

### 2.1. Classification of Meteorites

Meteorites are of special interest for some already mentioned reasons (e.g., laboratory analysis, leading to accessible information of the solar system history). This makes those objects special for scientific research, and classification schemes of meteorites were proposed. Basically, meteorite classification terminology follows geological mineral grouping. Meteorite mineralogy is complex since 275 mineral species have been reported so far [36]. Nevertheless or exactly because of this diversity, this proxy is a diagnostic for the origin of the sample. Broadly screened, meteorites are classified into three major classes [36]:

Iron: composition principally of pure metallic nickel-iron (sensitive to oxidation).

Stony: principally silicates or rocky meteorites (requires careful laboratory analysis to determine extraterrestrial origin).

Stony-iron: a mixture of the previous two classes.

**Figure 2:**
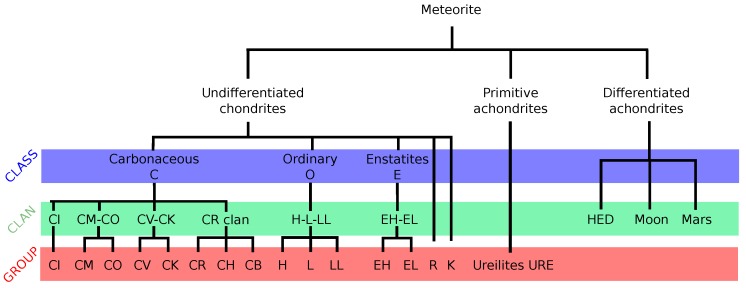
Classification of meteorites. This figure is adapted from the classification scheme, as shown by Weisberg, McCoy and Krot [52].

In addition, meteorites are grouped into falls and finds. Falls refer to an observed landing, and a find is a meteorite discovery. A more detailed scheme of classification of meteorites is sketched in Figure 2. Special emphasis is given to undifferentiated chondrites, especially carbonaceous chondrites (C chondrites), to study organic chemistry within these types of meteorites. CI chondrites (C for carbonaceous and I for the Ivuna meteorite) are thought to be the most primitive meteorites in terms of mineralogy [50]. C chondrites contain up to 4 wt% (percentage by weight) carbon-bearing organic matter [35]. Additional help with classification is provided from cosmochemical meteoritic isotopic analysis [51]. Bulk H, C and N abundances were determined, as assessed mostly in insoluble organic matter in meteorites.

An important issue within meteoritic research is the question of the parent body of a given meteorite, as meteoroids are generally formed by collision of two celestial bodies. Meteorites are well-described that originate from asteroid parent bodies (e.g., HED (howardite–eucrite–diogenite) meteorites or Chelyabinsk), from planets (e.g., Mars) or from the Moon (e.g., lunar meteorites) [47]. To the best of our knowledge, no meteorite was ever directly found with a certain cometary origin, although properties of meteorites with a putative cometary origin were studied [53,54,55,56]. Nevertheless, interplanetary dust particles were observed in the Earth’s atmosphere [57]. These results could be relevant since interstellar dust is directly connected to cometary material [58]. Furthermore, dynamical studies and meteor observations indicate a continuum between dark asteroids and comets and conclude that there should be a small fraction of the ≈30,000 meteorites that originate from comets [59]. Differences between CI1 chondrites and cometary nuclei could be ascribed by recent space missions, which were recorded basically from Stardust data, a NASA mission to collect cometary dust samples [59]. It has been suggested that type 1 (and maybe type 2) C chondrites are the best candidates for being cometary meteorites. In direct comparison, CI meteorites may have a D/H (deuterium over hydrogen) ratio that is lower than the average values of the D/H ratios that have been measured for comets [60,61].

Additionally, meteoritic composition is influenced by shock processes (e.g., by impact, collision during meteoroid formation), thermal metamorphism (e.g., by short-lived radionuclide heat of decay) or aqueous alteration effects. These secondary effects are considered in meteoritic classification. By terminology, shock grades range from S1 (completely unshocked, up to 5 GPa, Giga Pascal) to S6 (very strongly shocked, 75–90 GPa). Thermal metamorphism and aqueous alteration of meteoritic bodies is categorized by different petrologic types (types 1–6). Type 1 and 2 meteorites are affected by aqueous alteration at low temperatures with increased alteration in type 1 objects with respect to type 2 meteorites. Aqueous alteration is subclassified from type 2.7/2.8 (least altered) toward type 2.0 (most altered) meteorites. Types 3–6 reflect increasing thermal metamorphism (type 6 meteorites were most affected by in situ parent body heating, whereas type 3 meteorites were fairly unaltered [47]).

### 2.2. Chondrules, CAIs, Cosmochemistry

The chondrite subclass of stony meteorites is characterized by globules of once-molten material that quickly solidified. These globules are called chondrules and are remnants of the early protoplanetary disc processes. Their composition is usually similar to that of the Sun and contains silicate minerals, e.g., olivine (Mg, Fe)${}_{2}$SiO${}_{4}$. Chondrules are attractive, weakly-processed samples for isotopic ratio determinations. Secrets about the early solar system may be unlocked to gain insights into the composition of the interstellar dust. Putatively, even older are the calcium-aluminum-rich inclusions (CAIs), submillimeter- to centimeter-sized light-colored calcium- and aluminum-rich inclusions that are found within C chondrites. Probing CAIs via lead (Pb-Pb) isotope radiometric dating (using radioactive clocks) represents a sensitive measure for geological time scales. For the Northwest Africa 2364 (NWA 2364) meteorite, an age of 4568.22 ± 0.17 million years has been determined, which was inferred as the beginning of the formation of the planetary system, meaning that this epoch could be interpreted as the age of the solar system [62]. This field of measuring meteoritic isotopic signatures is known as cosmochemistry. Chemical cosmology can be interpreted as elucidating the formation of the solar system with the help of chemical probes. Cosmochemistry is also used to track the spatial/astronomical origin of a meteoritic sample within the solar system, e.g., via the determination of the deuterium/hydrogen ratio (D/H) [63]. Generally, it is important to match data from different techniques (radio astronomy, meteoritic laboratory analysis, computational modeling). One example of an unclear particular astronomical origin is the NWA 7325 meteorite, which has been extensively discussed to originate from Mercury [64,65,66].

In addition, chondrites might also be a source of interstellar molecules. How are these complex organic molecules formed within a chondritic body? Did they form within a meteoric body or outside from a chondrite? Are there any interactions between organic molecules and minerals? However, the source of organics within chondritic compartments and their interaction with minerals is not well understood yet. Further studies both from analytical meteoritic chemistry and laboratory studies on interstellar ice analogs are needed to understand better the origin and formation of ancient complex organic molecules, also in terms of spatial information.

## 3. Organic and Metalorganic Material in Meteorites

Searching for organic matter is mostly done within carbonaceous chondrites (C chondrites) [67]. C chondrites contain up to 4 wt% organic matter carbon [35]. This field of research may possibly be connected to origin of life/astrobiological questions. The organic matter contained in chondrites may have been an essential part of the organics needed as seeds for prebiotic chemistry. The Murchison meteorite, an observed fall of ≈100 kg in 1969 in Australia, influenced significantly the studies on meteoritic organic material [68]. Early investigations on the Murchison meteorite by well-equipped laboratories was motivated at that time also in the context of Moon return samples from the Apollo missions, a NASA program dedicated to manned lunar landing. Over almost 50 years, Murchison has become a valuable extraterrestrial organic reference material, which has been analyzed by modern analytical techniques.

Organics in meteorites are known to be present in various forms. Meteoritic organic matter is traditionally grouped into soluble and insoluble organic matter (SOM and IOM). Soluble organic matter represents a small fraction of free molecules, including prebiotically-relevant compounds such as amino acids or fatty acids. This fraction is defined to be soluble in organic solvents and/or water. The major part (≈70%) of the meteoritic organic matter is described as being insoluble and refers to a highly cross-linked aromatic chemical network or macromolecules whose structure resembles the one of a terrestrial kerogen [69]. Beside studying SOM and IOM separately, several research groups investigated the transition between SOM and IOM [44,70].

### 3.1. Insoluble Organic Matter

**Figure 3:**
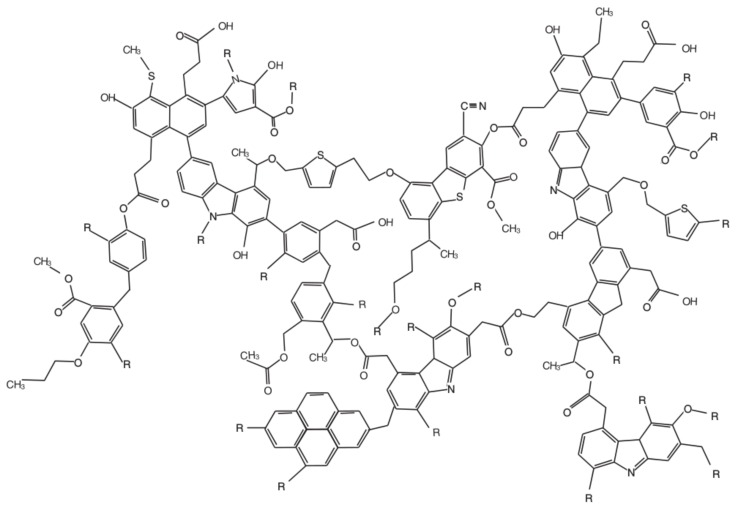
Model of the molecular structure of Murchison insoluble organic matter. The figure is adapted with permission from John Wiley & Sons, Inc., Hoboken, New Jersey, United States [76].

Studying insoluble organic matter requires extensive analytical sample preparation. A meteoritic specimen is suspended in a mostly organic solvent to separate soluble from insoluble organic matter. In the next step, the residue is considered as insoluble organic matter and gets dissolved in an HF/HCl mixture to remove minerals [71]. Subsequently, IOM is often thermally and chemically degraded (via flash pyrolysis and RuO${}_{4}$ oxidation to release aromatic and aliphatic moieties). Remaining organic compounds are analyzed via gas chromatography mass spectrometry (GC-MS) [72]. Alternatively, nuclear magnetic resonance spectroscopy (NMR) [69,73] or Raman spectroscopy [74,75] is used to elucidate meteoritic IOM. IOM is generally characterized as a highly macromolecular chemical network of high chemical aromaticity. Derenne and Robert proposed a chemical model structure for insoluble organic matter of Murchison (Figure 3) [76]. Despite a high degree of unsaturation/aromaticity, incorporation of heteroatoms (oxygen, nitrogen, sulfur) can be observed. Chemical parameters were described as H/C = 0.70, O/C = 0.22 and N/C = 0.03 [76]. Additionally, Raman spectroscopy studies on IOM provide information on the thermal metamorphism of meteorites [77,78].

### 3.2. Soluble Organic Matter: Amino Acids and Beyond

Soluble organic matter in meteorites, often called free organic compounds [79], is of high interest, especially in prebiotic focus [80]. Herein, amino acids are perhaps the most often discussed compound class in carbonaceous chondrites. Special care is taken with the issue of terrestrial contamination [81,82,83], e.g., on carbon and nitrogen stable isotope composition [84]. Carbon isotopic measurements are routinely used to test organic target molecules for extraterrestrial origin [85].

#### 3.2.1. Amino Acids

Amino acids are the building blocks of life within proteins. In addition, these molecules were found within astronomical environments, namely comets [38]. The Martian meteorite Allan Hills 84001 (ALH 84001) attracted intense media attention in 1996. U.S. president Bill Clinton gave a speech about potential life on Mars [86]. Interpretations have been based on studies of ALH 84001, which was examined for organic material and fossils [87]. Subsequent conclusions deciphered that the detected fossils and polyaromatic hydrocarbons are probably artifacts or terrestrial contamination, respectively [88]. Isotopic measurements of ${}^{14}$C content of ALH 84001 organic matter could be related to Antarctic terrestrial contamination [89]. The analysis of amino acids in ALH 84004 also showed that these amino acids appear to be terrestrial in origin [90]. Nevertheless, the search for amino acids, the building blocks of life, are an ongoing highlight within general meteoritic research. Analysis of fresh Murchison meteorite samples revealed the presence of both proteinogenic and non-proteinogenic amino acids, in concentration ranges of ≈60 ppm [79,91]. Ninety two amino acid structures could be identified in meteorites to date [27,28,29,92,93].

#### 3.2.2. Nucleobases

Nucleobases represent key molecules in prebiotic chemistry and were found in the Murchison meteorite, as well [85]. The nucleobases pyrimidine and purine are essential for terrestrial organisms, which depend on nucleic acids (RNA and DNA) to encode genetic information, a crucial biotic process in all known forms of life. In addition to Murchison, nucleobases were found later in another eleven meteorites, mostly carbonaceous chondrites [94]. Interestingly, Almahata Sitta #4, an ureilite meteorite, was reported to contain the nucleobase adenine, as well [94].

#### 3.2.3. Sugars

Additionally, other prebiotically-relevant molecules were observed within meteorites. Studies on the carbonaceous chondrites Murchison and Murray revealed the presence of polyols (sugars and derivatives) [95] in comparable amounts to amino acids. Sugars are, together with nucleobases, essential building blocks of nucleic acids, representing important biomolecules. Nevertheless, phosphorylation reactions are still one of the critical and not yet understood crucial steps in modern prebiotic chemistry [96,97,98,99,100].

#### 3.2.4. Carboxylic Acids

Next to proteins or nucleic acids, carboxylic acids are of significant biochemical importance. Vesicle formation, membrane bilayer structures and finally the build-up of biological minimal cells are mainly dependent on amphiphilic molecules, like carboxylic acids/fatty acids. Fatty acids represent a compact store of energy in cell membranes. Therefore, attraction was focused on the analysis of carboxylic acids within meteorites [101,102]. Carboxylic acids are known to reach concentrations of ≈300 ppm within carbonaceous chondrites and represent the most abundant organic species in meteorites [47].

#### 3.2.5. Chirality

The concept of chirality is an important measure for living systems by differentiating D and L enantiomeric conformers. D and L labels originate from the Latin words *dexter* (on the right) and *laevus* (on the left). The enantiomer that rotates plane-polarized light clockwise (+) was arbitrarily defined as a D enantiomer.

In other words, homochirality represents a signature of life. To our present-day knowledge, nature uses almost exclusively L-amino acid and D-sugar enantiomers to incorporate these biomolecular building blocks into protein or nucleic acid chemical machineries within living systems [103]. This phenomenon attracted many people within the field of origin of life studies. Biotically-relevant enantiomeric excess of both amino acids [104] and sugars [105] was studied within meteorites. The amino acid isovaline was found to have non-terrestrial L-excesses of up to 18% in the Murchison meteorite [92,106]. Furthermore, sugars were found in excess in their D enantiomeric form in meteorites [105]. Anyhow, the origin of homochirality in chiral biotic molecules is still under debate. Many theories have been presented, ranging from “by chance” versus “determinism”, including chemical models like autocatalysis or physical models like enantiomeric amplification as triggered, for example, via circularly-polarized electromagnetic radiation [107,108,109,110,111,112,113].

Generally speaking, soluble free organic compounds can be analyzed by two different analytical approaches, by means of targeted or non-targeted analytical strategies. The above described examples represent targeted analyses, in which a precisely-defined goal exists, as the search for amino acids within a Martian meteorite to gain insights into the probability of existence of Martian life. Within this fairly rigid hypothesis-driven research, important latent analytes may be overlooked. In the following, motivations for non-targeted analytical methodologies and their power within meteoritic organic matter studies will be given.

### 3.3. High-Resolving Chemical Analytics

A critical parameter in studying in-depth meteoritic organic matter is analytical instrumentation. A significant increase in instrumental quality has been observed over the last few decades. Huge steps forward in terms of analytical sensitivity, accuracy, precision and resolution have been made. Special emphasis is put here on high-resolving chemical analytics. Modern equipment, like high-resolution mass spectrometry (MS) and nuclear magnetic resonance spectroscopy (NMR), pushed organic astrochemistry significantly forward. The increase of sensitivity, accuracy and resolution by orders of magnitude within analytical instrumentation enabled previously “unseen” astrochemical insights, like the detection of ribose within interstellar ice analogs by two-dimensional gas chromatography coupled to mass spectrometric detection (GCxGC-MS) [114].

#### 3.3.1. In-Depth Compositional Profiling: Ultrahigh-Resolving MS

Mass spectrometers act like fine scales that “weigh precisely masses of molecules” (measuring mass-over-charge ratios, *m*/*z*). Ultrahigh-resolving instruments, like Fourier transform ion cyclotron resonance mass spectrometry (FT-ICR-MS), represent some of the most powerful tools to allow insights into complex chemical spaces. FT-ICR mass spectrometry offers the highest mass resolving power and mass accuracy among all types of mass spectrometers [115,116]. This enables the study of complex mixtures. Frequency-based measurements, like in FT-ICR-MS, result in extremely high mass resolving power *R* (>10${}^{6}$) and mass accuracy (<200 ppb). Thus, molecules with mass differences less than the mass of an electron can be distinguished. Measured *m*/*z* signals are assigned to molecular compositions for further data processing.

In addition to ultrahigh-resolving FT-ICR-MS techniques, Orbitrap instrumentation is used more and more in astrochemical studies. In comparison to FT-ICR-MS, Orbitrap is lower in resolving power and mass accuracy by approximately one order of magnitude (*R* ≈ 10${}^{5}$ and mass accuracy ≈2 ppm) [117,118]. Investigations on refractory carbonaceous components of interstellar ice/pre-cometary ice analogs [119], simulated cometary atmospheres [120] or on meteorites were performed [121,122,123]. Striking results like the detection of nucleobases in meteorites were reported using high-end analytical methods [94]. Additionally, Orbitrap mass analyzers are also envisaged as space mission instruments to characterize in situ planetary environments [124].

A disadvantage of mass spectrometry is that only molecular formulas are derived out of measured *m*/*z* signals. No direct structural information is provided as a first hint. Anyhow, ideas on structural building blocks can be gained by tandem MS (MS${}^{n}$) [125] or ion mobility MS (IMS) [126].

Data-analytical methods were developed to gain insights into chemical structural properties. Senior reported in 1951 graph-theoretical derivations to elucidate chemical structural information out of molecular formulae (via the cyclomatic number) [127]. Basic assumptions here are the similarity between molecules and graphs (networks). This mathematical concept allows for applying graph-theoretical rules to molecular systems. Based on Senior’s work, many applications were reported to describe structural characteristics for given elemental compositions, e.g., the seven golden rules of Kind and Fiehn [128]. In addition, Pellegrin presented in 1983 a second, similar approach to Senior’s, on the nitrogen rule and degree of unsaturation of organic molecules [129]. This approach has been applied further to describe chemical properties on aromaticity via the aromaticity index [130] or the aromaticity equivalent approach [131].

#### 3.3.2. Data-Driven Astrochemistry

Ultrahigh-resolving chemical analytics is directly connected to high-dimensional datasets. Schmitt-Kopplin et al. reported a high chemical diversity in meteorites [20]. Chemical information of thousands of individual components out of a complex organic mixture was assessed from diversely-classified meteorites [20,132,133,134,135]. Tens of thousands of different molecular compositions and likely millions of diverse structures were observed in solvent extracts of pristine carbonaceous meteorites [20].

**Figure 4:**
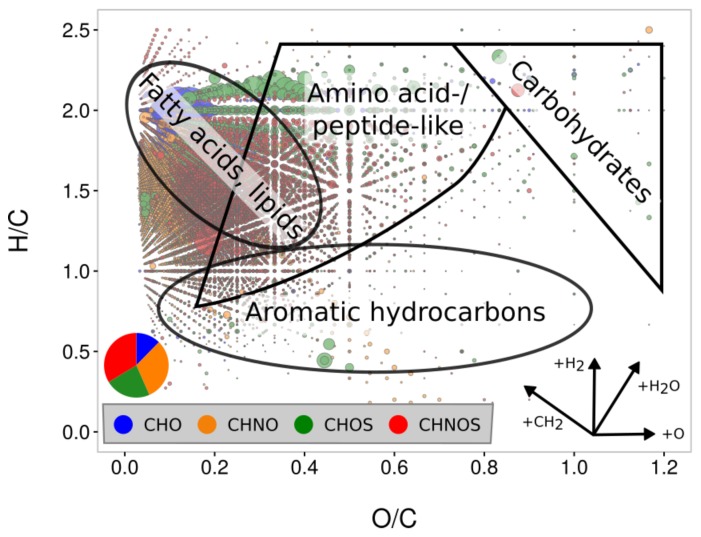
Van Krevelen diagram of Murchison soluble organic matter. O/C versus H/C is plotted for negative ionization ESI-FT-ICR-MS methanolic soluble organic matter of Murchison. The bubble size is normalized to mass spectrometric intensity. Chemical subspaces: CHO (blue), CHNO (orange), CHOS (green), CHNOS (red) with its respective partitions. Approximately 15,000 molecular formulae are shown [20].

Data analytical tools are required to extract information out of these high-dimensional and complex chemical datasets. Dealing with thousands of detected signals, visualizing data is a crucial first step to evaluate chemical analysis. A moderately simple, but powerful approach to plot data is a van Krevelen diagram [136]. Atomic ratios, mostly oxygen/carbon versus hydrogen/carbon, are plotted to gain chemical information on complex high-dimensional compositional spaces. Figure 4 shows a van Krevelen diagram of Murchison soluble organic matter, highlighting the presence of a large number of organic molecules. Approximately 15,000 molecular formulae are visualized. This representation enables one to gain information regarding chemical classes at first sight [20]. Fatty acids/lipids, aromatic hydrocarbons, amino acids or sugar-/carbohydrate-like compounds were observed. In addition, this representation depicts information on homologous series, as revealed from experimental FT-ICR mass spectrometric data. Differentiation of the complex organic mixture into chemical spaces CHO, CHNO, CHOS and CHNOS is enabled. Variability in the degree of unsaturation (via H/C ratio) and oxygenation (O/C ration) can be deciphered. van Krevelen diagrams enable also visual comparison of different samples regarding their chemical similarities.

**Figure 5:**
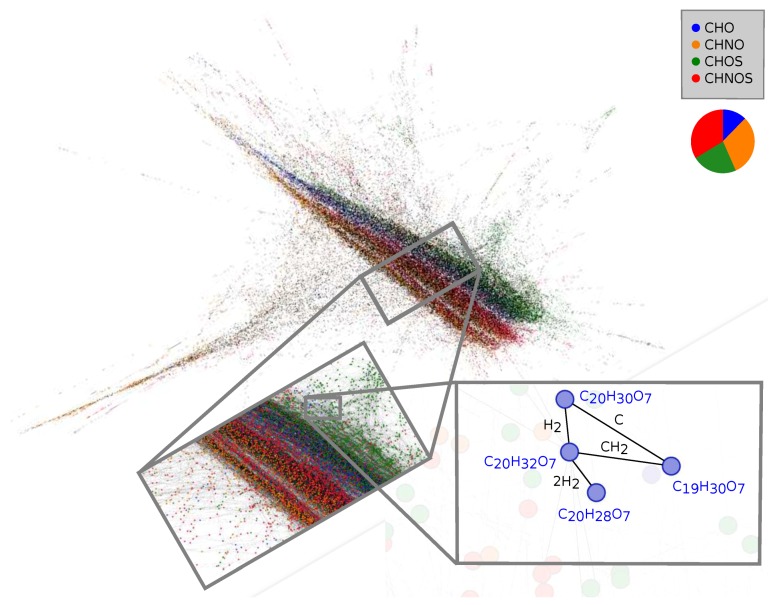
Mass difference network of Murchison soluble organic matter, as analyzed from negative ionization ESI-FT-ICR-MS data. The network was visualized via Gephi software [141], using the Force Atlas2 layout algorithm. Chemical subspaces: CHO (blue), CHNO (orange), CHOS (green), CHNOS (red) with its respective partitions. Approximately 15,000 molecular formulae are shown [20].

Another data-driven analytical approach to gain insights into complex organic chemical spaces is mass difference network analysis [137,138,139]. Herein, nodes represent experimental *m*/*z* values, and edges (connections within the network) represent exact mass differences, which are equivalent to a net molecular formula of a chemical reaction. Chemical diversity is visualized. Mass difference networks allow for studying unknown chemical substances (“chemical dark matter”). Additionally, chemical pathways can be studied within reaction sequences only with the help of molecular formulas [140]. Figure 5 shows a mass difference network of soluble organic matter of Murchison, as computed from negative ionization ESI-FT-ICR-MS experiments. Approximately 15,000 molecular formulae are depicted to get information on the global distribution of chemical spaces CHO, CHNO, CHOS and CHNOS [20]. In this analysis, edges (reaction-equivalent mass differences) are represented by fundamental astrochemical building blocks (e.g., H${}_{2}$, CH${}_{2}$, N${}_{2}$ or SO${}_{2}$). Sixty-six percent of all mass differences are C-, H- and O-bearing molecular formulas. Thus, interconnections among heteroatomic chemical subspaces (e.g., CHO-CHNO transitions) are not widely present, and heteroatomic chemical spaces remain to be fairly separated. A zoomed-in picture of CHO-bearing molecules, including their respective connected mass differences, is depicted (Figure 5). This compartment within the complex chemical network illustrates basic aliphatic carbon chemistry within Murchison soluble organic matter. Herein, an exemplary chemical reaction of the shown network in Figure 5 is:

$$\begin{array}{c} C_{19}H_{30}O_{7}+CH_{2}\to C_{20}H_{32}O_{7} \end{array}$$

Data-driven mass difference network analysis is sensitive to experimental mass accuracy. Routinely, mass accuracy ranges of ±0.1–0.2 ppm are required to gain valid results via mass difference network analysis. This makes this method still challenging to directly apply Orbitrap experimental data. Nevertheless, FT-ICR mass spectrometry enables valid experimental data for studying comprehensive chemical characteristics out of high-dimensional chemical space datasets [138,139].

Data science profits from global data analysis by implementing statistical, ensemble-related data treatment to understand and analyze general phenomena with data [142]. A major aspect of motivation in data-driven science is that results and subsequent interpretations of scientific problems should be independent of the experimenter’s hypothesis, but only be based on observed data [143]. High-resolving chemical analytics of meteorites, including thousands of data per sample, allows for moving forward in analyzing meteoritic organic matter with the help of data-driven analytical methods such as network approaches or machine learning techniques [20,144].

The application of multivariate statistical methods (e.g., principal component analysis (PCA) or partial least squares analysis (PLS)) will allow one to extract significant features out of a multiple complex feature spaces with thousands of compounds each [145,146]. Therefore, discriminant molecules can be identified that differ in relative abundance between several samples, even out of complex chemical mixtures.

#### 3.3.3. Structural Chemical Information: Organic Spectroscopy

**Figure 6:**
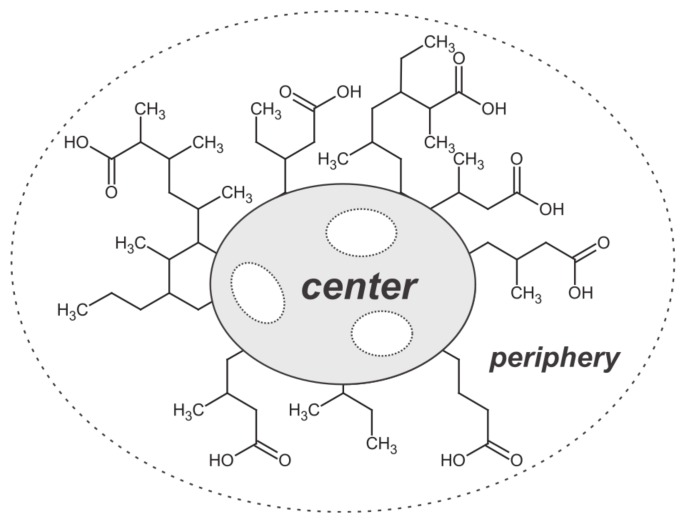
Model of the molecular structure of Murchison soluble organic matter. The figure is adapted with permission from John Wiley & Sons, Inc., Hoboken, New Jersey, United States [150].

Complementary to comprehensive compositional information, spectroscopic techniques, like NMR, infrared (IR) or Raman spectroscopy, provide insights into chemical functionalities. IR spectroscopy studies revealed the presence of carbonyl compounds (aldehydes, ketones) in solvent extracts of the Murray [147] or the Orgueil meteorite [148]. Compositional diversity in meteoritic IOM was also revealed by IR spectroscopy [149]. Additionally, Raman spectroscopy represents an important tool within the characterization of insoluble organic matter [74,75]. NMR spectroscopy was used to characterize both insoluble [69,76] and soluble organic matter [150]. Hertkorn et al. proposed in a non-targeted NMR approach a model structure for soluble organic matter for Murchison (Figure 6) [150]. Aliphatic methyl, methyl in the β-position to carboxylic groups and carboxylic groups were reported in a ratio of 12:2:7.

#### 3.3.4. Insights on Chemical Isomers: Chromatography

In addition to compositional and structural information, differentiation of chemical isomers is probed via analytical separation techniques. Capillary electrophoresis (CE) analyses revealed insights into amino acid chirality [151]. Gas chromatographic studies (GC-MS) detected sugars [95], diamino acids [152] or nucleobases [85] in meteorites. Results from two-dimensional GCxGC-MS methods revealed the challenging detection of ribose out of a complex mixture including various conformers within interstellar ice analogs [114] and suggest a pathway for prebiotic ribose formation, the central molecular subunit in RNA.

By combining state-of-the-art chemical analytical techniques, probing compositional complexity, chemical structural information and isomeric specific data, chemical complexity can be studied in great detail. The Schmitt–Kopplin group developed and optimized for more than a decade ultrahigh-resolving compositional FT-ICR-MS [20], structural NMR spectroscopic [150] and chromatographic [20,132] analyses on complex meteoritic organic matter in a comprehensive non-targeted analytical approach.

### 3.4. Targeted versus Non-Targeted Analysis

The molecular diversity of extraterrestrial organic matter in carbonaceous chondrites represents a chemodiversity hotspot. As shown numerous times for Murchison, which is considered as the carbonaceous meteoritic reference material, likely millions of organic compounds were found [20]. Chemical analysis were performed by means of both targeted [80,95,152,153] and non-targeted [20,150,154] methodologies. Both approaches complement each other and converge in combination for gaining as much information on the studied scientific system as possible. At first sight, the non-targeted methodology can be seen as a top-down profiling/screening approach that profits from targeted complementary results (and vice versa), whereas the targeted approach elucidates specific systems in-depth in a bottom-up manner. In a non-targeted approach, all analytes are globally profiled within maximum analytical possibilities without being biased or constrained by a hypothesis in order to gain comprehensive information in a top-down manner. As such, the holistic non-targeted analyses of meteoritic soluble organic matter revealed the comparison of extraterrestrial and terrestrial molecular diversity [20,33,150].

#### 3.4.1. Thoughts on Comprehensive Chemical Analysis

**Figure 7:**
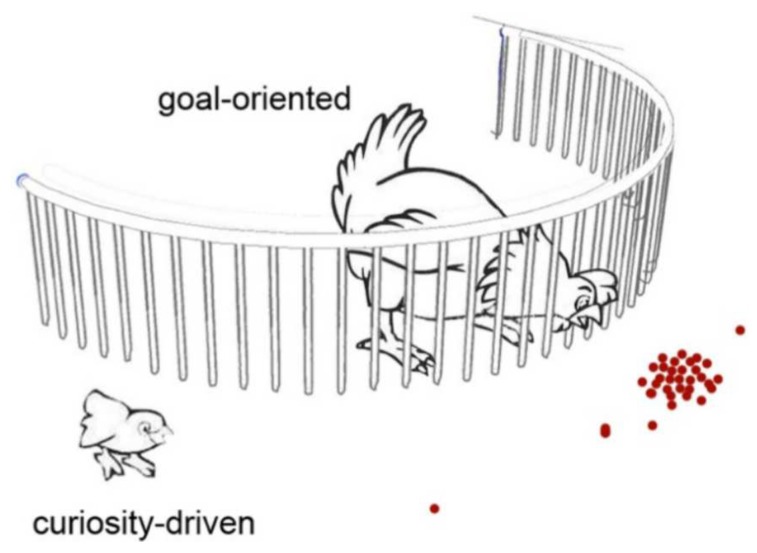
Curiosity-driven research. This figure is adapted with permission from ©The Nobel Foundation, Nobel lecture of Laureate Theodor W. Hänsch, Stockholm, 8 December 2005 [156].

Every analytical chemical method starts with defining a hypothesis prior to analysis. Targeted analysis mostly fixes these hypotheses with respect to a set of defined molecules that should be detected or quantified. Non-targeted approaches define the analytical goal by describing chemical processes, seeking a more global chemical description of a sample or studying interactions within a complex chemical network including all analytes. In short, the term target is a synonym of the chemical compounds within a sample. Non-targeted methods profit from not overlooking specific analyte molecules. In non-targeted methods, hypothesis are set based on data, not on a specific interest of the analyst [155]. Figure 7 illustrates the power of curiosity-driven research, motivated for not defining too strictly analytical targets. In contrast, targeted methods are able to dig deeper for mechanistic studies of single-analyte molecules. Therefore, both analytical strategies enable a powerful complementary set for analyzing chemical mixtures.

Generally speaking, in an abstract manner, the appropriate choice of an analytical method to study a sample of interest is not so trivial: it can even become a philosophical (epistemologal) problem. Probing a sample is motivated by the specific interest of an analyst. For example, one is interested in the absolute or relative abundance of one specific molecule in a sample, e.g., the absolute abundance of a specific amino acid within a Martian meteorite. Another type of analyst’s interest could be the probing of intensity distributions of fatty acid homologue series within a sample of interest. At this second level, interactive effects for a set of single fatty acid molecules can be deciphered due to the simultaneous detection of chemically similar molecules. When moving one abstract layer forward, the interest of an analyst can be described as comprehensive probing of a certain sample of interest. Herein, all molecules of a sample are defined as actual analytes. In fact, in the third case, samples will be studied, including their ingredients’ interactions in total. Analytes are not treated as isolated systems within the sample of interest. A general sketch of different fundamental analytical strategies can be summarized as follows. 

TargetedAnalytes are single molecules
→ Quantification/monitoring of single molecules/studying molecules on a mechanistic levelSemi-targetedAnalytes are a set of molecules
→ Relationships of chemically similar moleculesNon-targetedAnalytes are all present molecules (in theory)
→ Information on as many molecules and their in-between interactions/screening of global chemical spaces

#### 3.4.2. Non-Targeted Analysis in Practice

The analytical scenario of a global, complete screening of both analytes and analytes’ interactions represents an ideal that cannot be realized practically. It is obvious that not all molecules can be simultaneously sampled within one analytical method. Instrumental constraints limit the coverage of analytical targets within an experiment. For instance, in mass spectrometry, a certain number of molecules is discriminated by the ionization mode (e.g., apolar molecules in electrospray ionization (ESI)). Another example is the analytical technique gas chromatography, which specifically focuses on volatile chemical compounds.

When thinking about targeting a chemical sample as holistically as possible, the analytical matrix sets the role of the chemical analytes, which goes in hand with several problems. In targeted analysis, the interfering effect, which can confound a target peak, is called a matrix effect [155]. Methods were developed to balance out the matrix when targeting single compounds, e.g., the standard addition method [155]. However, in non-targeted strategies, the matrix effect cannot be elucidated so easily and represents a present-day problem within this kind of analytical approach [157]. Therefore, the intention of completely probing a chemical system is a fundamental problem of experimental research. Results and interpretations are always dependent on “what you see”, meaningful scientific conclusions are dependent on detection specificity and sensitivity. Therefore, what is actually a global chemical profile? In practice, non-targeted, comprehensive analysis is dominated by high-dimensional big data experiments (data-driven astrochemistry), which were discussed above.

### 3.5. Combining Experimental and Computational Techniques

Astrochemistry deals with molecular complexity and diversity [1]. The presence of ≈200 molecules detected in interstellar and circumstellar media was not always expected by the scientific community [2]. This remarkable chemical space is not trivial to understand regarding the formation and stability of its organic molecules under highly energetic and out of equilibrium conditions in astronomical environments. The combination of quantum chemical simulations with observational results is powerful to understand astrochemical systems [158]. With the significant increase in computational power over the past few years, large molecular systems have been probed via molecular simulations.

As an example, interstellar grain prebiotic chemistry was computed by means of interactions of glycine and alanine on polycyclic aromatic hydrocarbon flakes [159]. Enhanced stability of the carboxyl group by chemisorption could be found, and implications for enantioselection were proposed. This work is one example for possibly important results in prebiotic chemical evolution in absence of experimental data. Nevertheless, the power of quantum chemistry here is to help laboratory experimental modeling and observational studies by suggesting theoretical data, like here on adsorption energies within this relevant interstellar prebiotic system. As another example, quantum chemical studies could provide insights into the lack of detection of interstellar anions [160]. This work underlines the importance of valence and dipole-bound excited states in the detection of anionic species in the ISM. These results may help, in addition to future laboratory data work, to detect interstellar anions.

In the meteoritic context, the interplay between experimental and computational techniques enabled structural analysis of organomagnesium compounds out of complex chemical mixtures [161]. Therein, information on chemical structures was accessed via a combination of fragmentation mass spectrometry and DFT/and second-order Møller–Plesset perturbation theory (MP2) studies. Due to the complexity of the chemical mixture, traditional structural elucidation techniques, such as NMR or IR spectroscopy, could not have been applied in this work. The complementary approach of experiment and theory represents a powerful alternative therein.

In terms of computational methods, two methods are presented here. Specific emphasis should be put here on density functional theory (DFT) and second-order Møller–Plesset perturbation theory (MP2).

Density functional theory (DFT) is the current workhorse in theoretical chemistry determining electronic structures. Different from traditional ab initio quantum chemistry concepts like Hartree–Fock theory (HF), DFT replaces Ψ by the electron density function ρ to describe the quantum mechanical system and its energy [162]. DFT often succeeds in computational costs over ab initio quantum chemical methods and can be therefore adapted to medium-large molecular system (<50 atoms). Sensitive to the accuracy of DFT results is the choice of the functional and the basis set regarding the respective chemical system to compute.

Møller–Plesset perturbation theory (MP2) represents an ab initio quantum chemical method, describing a system’s energy by the many-body wave function Ψ. Have an advantage with respect to Hartree–Fock theory (HF) routines, MP2 computations include electron correlation effects, which increases the accuracy in describing the electronic properties of a chemical system [163]. Practically, MP2 computations are more accurate than DFT simulations, but are also computationally much more expensive. Therefore, MP2 simulations on moderately large molecular systems are not fully practicable.

In conclusion, the interplay between experimental and computational methods is powerful within organic astrochemistry studies. Sparse observational data or the degree of complexity of targeted chemical network require help from quantum chemistry methods. Increasing computational power enables nowadays a quantum-mechanical description of challenging complex molecular systems, including a fairly high number of atoms.

### 3.6. Comprehensive Chemical Profiling of Meteoritic Organic Matter

Studying meteoritic soluble organic matter via non-targeted analytical strategies (data-driven astrochemistry) represents a powerful tool to probe astrochemical complexity in a broad molecular range (100–1000 amu, atomic mass units). To date, thousands of individual components have been profiled within complex organic mixtures from diversely-classified meteorites [20,144,150]. Likely millions of diverse structures were observed in solvent extracts of pristine C chondrite meteorites [20,150]. This suggests that extraterrestrial chemistry is extremely active and rich.

The extreme richness in chemical diversity of meteoritic soluble organic matter offers information on the meteoritic parent body history. Hints about heteroatom incorporation and its chronological assemblies, shock and thermal events can be given by advanced data analytical methods of these correlated high-dimensional datasets [21]. Heteroatomic organic and metalorganic molecules (e.g., N-, S- or Mg-bearing compounds) play an important role in the description of chemical evolution. It could be shown that coupled high-resolving instruments in combination with sophisticated data analytical methods (e.g., molecular networks) help in expanding our knowledge in astrochemistry toward higher molecular masses and complex molecular structures [21].

#### 3.6.1. Nitrogen Chemistry

**Figure 8:**
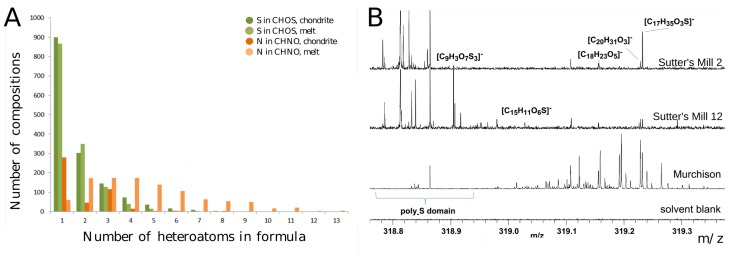
Role of nitrogen and sulfur chemistry. (**A**) The thermally- and shock-stressed Chelyabinsk showed high numbers of nitrogen atoms within CHNO molecular formulas [133]; (**B**) The extremely thermally-altered Sutter’s mill reflects a loss in organic diversity, but an increase in the polysulfur domain, as compared to Murchison [21,166]. The figure is adapted with permission from The American Association for the Advancement of Science, Washington, D.C., United States [133,166].

Heteroatomic organic molecules were found to play an important role in the description of chemical evolution. The thermally- and shock-stressed Chelyabinsk (LL5 chondrite, LL stands for low (total) iron, low metal contents) [133] showed high numbers of nitrogen atoms within CHNO molecular formulas, relative to other L-type meteorites with lower shock grades, especially in the melt region (Figure 8). Analogous concordance could be also observed for Soltmany (L6 chondrite) [132] Novato (L6 chondrite) [164] and Braunschweig (L6 chondrite) [165]. L and LL ordinary chondrite meteorites are similar in their petrologic composition.

#### 3.6.2. Sulfur Chemistry

The extremely thermally-altered Sutter’s mill (C-type, C stands for carbonaceous) reflects a loss in organic diversity (positive mass defect region), but an increase in the polysulfur domain (negative mass defect region), as compared to other CM2-analyzed falls (C stands for carbonaceous and M for the Mighei meteorite, Figure 8) [21,166]. Sutter’s mill soluble organic matter specifically exhibits many signals in the mass range of 318.75–319.0 amu, corresponding to oxygen-rich and multiple sulfur-containing molecules.

### 3.7. Metalorganics in Astrochemistry

Metalorganic molecules have rarely been discussed in astrochemistry [167,168]. Scientists focused mainly on studying either organic material (discussed above) or minerals [169] within astronomical environments. Interestingly and contradictory to the lack of research, they are supposed to represent key intermediates for organic evolution.

Particularly as reported for interstellar medium reactions, iron interactions with polyaromatic hydrocarbons (PAH) are discussed. Nevertheless, no organometallic species could be observed within the ISM. The formed Fe-PAH complexes have been proposed to influence the growth of PAH in evolved star envelopes and influence therefore the formation of aromatic organic molecules within these astronomical environments [170,171]. Additionally, Fe${}^{n+}$ has been proposed to be involved in the destruction/formation of CO [172] and formation of HCO [172].

In meteoritic context, little work has been done on mineral-organic spatial associations, e.g., interactions between aromatics and carboxylic functional groups with phyllosilicates for Renazzo meteorite (CR2 chondrite), Murchison (CM2 chondrite) and Orgueil (CI chondrite) [173], suggesting modification of organic matter by clay-mediated reactions [174]. Additionally, associations between aliphatic CH and OH in phyllosilicates in the Tagish lake meteorite (C2-ung chondrite, ungrouped carbonaceous meteorite) were found [175].

Generally, metalorganic species are well-known from the classical organic laboratory to efficiently catalyze various reactions [176]. This stimulates research on astrochemical molecules within astrochemical environments. As indicated above, the origin and formation of early complex organic molecules within the meteoritic matrix is still unknown. One possible pathway might be the formation via metalorganic intermediate states. Studies on the hypothesis that the formation of soluble organic matter in meteorites is related to mineral aqueous alteration were performed previously [177]. Nevertheless, the problem of primordial complex organic molecule formation still remains largely unsolved. Thus, more extensive research on complex organic molecules formation and the role of metalorganic species therein has to be done.

Recently, metalorganic compounds were discovered in meteorites [161]. Ultrahigh resolution FT-ICR mass spectrometry was used to detect this CHOMg chemical space in meteorites. This special technique exhibits extremely high mass resolving power (*R* > 10${}^{6}$) and mass accuracy (<200 ppb) that were required to unambiguously distinguish the organomagnesium compounds (CHOMg) from CHNOS compositions [161]. A novel class of organomagnesium compounds (CHOMg) could be proposed as important links within organic and mineral chemical evolution. A hitherto unknown chemical class, dihydroxymagnesium carboxylates [(OH)${}_{2}$MgO${}_{2}$CR]${}^{-}$, could be observed in meteoritic solvent extracts. These CHOMg compounds could be related to thermal metamorphism in meteorites and fractionation processes, which might shed new light on our understanding of carbon speciation in meteoritic parent bodies at a molecular level.

In addition, dihydroxymagnesium carboxylates [(OH)${}_{2}$MgO${}_{2}$CR]${}^{-}$ were probed for decarboxylation on a theoretical level, by utilizing both MP2 and B3LYP-DFT computations [178]. This study asks whether recently introduced, astrobiologically potentially relevant dihydroxymagnesium carboxylates form Grignard-type reagent molecules via decarboxylative fragmentation. It discusses the challenge of forming OH-bearing Grignard-type reagent out of CHOMg precursors in non-catalytic, low energetic environments, but provides ideas on its experimental realization.

## 4. Relevance of (Metal) Organic Astrochemistry: Implications on the Origin of Life

Research on complex organic molecules within astrochemical environments is often connected to origin of life/astrobiological questions, like the search for the building blocks of life [179,180,181]. When searching for these building blocks, one would need to define what is life first. This represents an intrinsic problem within this scientific question itself, since the definition of life is a long ongoing challenging task [182,183,184]. Many prominent scientists, e.g., Erwin Schrödinger, have done research on the question of the definition of life [184]. While digging deeper into a guess to strictly define life in a physical manner, many problems arose [184,185,186]. As an example, Schrödinger reported the problem of negative entropy within a rigorous physical definition [184].

Generally, life can certainly be defined through a collection of characteristics. Due to enormous physical and chemical complexity, this methodology would be more profitable rather than the concept of a definition via strict mathematical derivations. Many definitions are biologically oriented, including basically four characteristics, as proposed by Schulze-Makuch and Irwin [182].

- metabolism
- growth
- reproduction
- adaptation to environment

Benner, Ricardo and Carrigan asked for “a common chemical model for life in the universe” [187]. For the astrochemistry community, a chemical way of defining life might be more intuitive. Benner, Ricardo and Carrigan listed the following parameters as fundamental requirements for life.

•  Thermodynamic disequilibriumassuming Darwinian evolution to be a progressive process and “that life actually does something”•  Bondingcovalent bond, e.g., C-C•  Isolation within the environmentthe Darwinian cycle can proceed only if it replicates itself in preference to others; compartmentalization•  Carbon-like scaffolding“machinery’s nutrients”•  Energetic patterns in metabolismno equilibrium, but energy transfer•  Solventefficiency of chemical reactions within the liquid phase

Independent of the exact definition of life, a common aspect is the need for organic molecules (nutrients) within a living environment. Therefore, the search for organic molecules in potential habitable environments is required as one fixed criterion for life. Second, energetic conditions (temperature, pressure, electromagnetic radiation) are also important to be profiled, since energetic patterns in metabolism are needed for living systems. In conclusion, the search for organic compounds as building blocks of life is necessarily related to their surrounding energetic conditions to ask for molecular local stability and energetic reaction patterns within a found local chemical network.

Organic molecular diversity in meteorites likely reflects an integrated temperature history of the respective bodies [133,164,165,166]. Cometary systems and primitive bodies are conserved at lower temperatures (both primitive meteorites, keeping their original chemical complex signature (e.g., CM2 chondrites) and meteorites that have been altered with temperature and/or aqueous alteration) [47]. Since most meteoroids undergo high energy gradients within astrophysical environments, especially during the formation of a meteoroid body, high energy local surface temperatures and pressures are involved when two celestial bodies collide. The presence of highly diverse extraterrestrial meteoritic organic matter seems to be in contradiction at first glance. Therefore, thermolabile organic molecules (e.g., carboxylic acids [188]) must be operative under a wide range of conditions. The following questions thus arise:

- Are intermediate states within chemical evolution required to preserve organic compounds?
- Can minerals stabilize organic molecules within geological time scales?

The interaction of organic matter and minerals, especially clay minerals, is known to play an important role in organic chemical evolution via catalytic effects in meteorites [174]. The importance of metal ions within chemical evolution has been previously suggested numerous times [189,190,191]. Metal ions may drive prebiotic reactions via free-radical reactions. In particular, their role in peptide formation was studied in clay environments by polymerization of alanine and glycine [192].

In biotic, living systems (end-members of chemical evolution), metal ions play essential roles in about one third of enzymes [193]. Therein, metal ions are significantly involved in biochemical electron flow processes in a substrate or enzyme or change the substrate molecular conformations via specific bindings [194].

**Figure 9:**
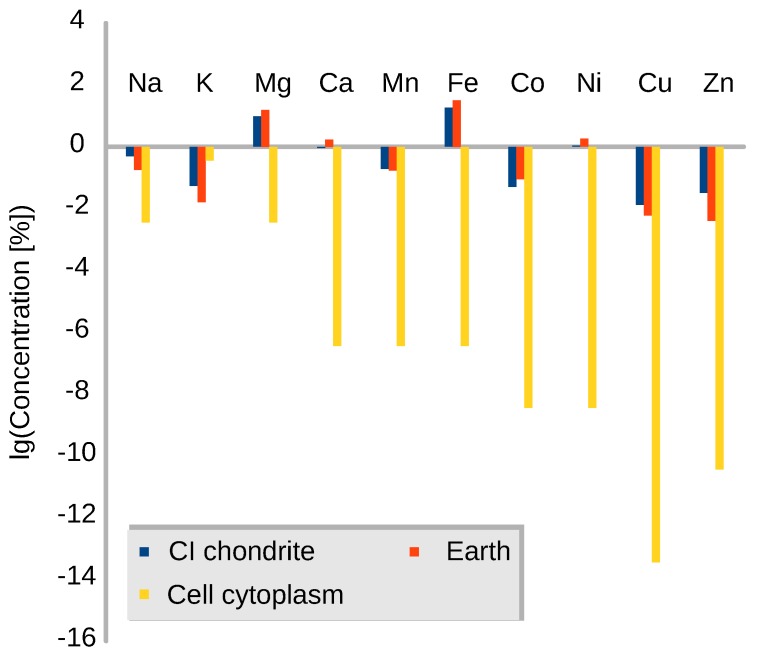
Metal distributions for meteorites, Earth and a biotic system. Data are adapted from Lodders for CI chondrites [50], McDonough for Earth [196] and Williams for cell cytoplasm [197].

Metal abundance distributions for several environments following the time line of chemical evolution (meteorites-Earth-biotic system, Figure 9) give insights into the relevance of different elements. Magnesium Mg and iron Fe are most abundant species for all three chemical systems, meteorites, Earth and biotic cell cytoplasm. In parallel, the significance of magnesium in traditional organometallic chemistry has been broadly studied and is well known [176]. Both abundance and organometallic affinity motivate probing magnesium-organic compounds within the context of chemical evolution. The finding of magnesium-bearing species (MgCN [195]) in proto-planetary nebulae argues for studying this research topic.

In addition, the recent finding of organomagnesium molecules (CHOMg) in meteorites argues for the importance of metalorganic species in the context of astrochemical/astrobiological research as these CHOMg compounds relate to meteoritic thermal processing [161]. In conclusion, these results on the chemistry of organomagnesium compounds have increased interest to further the role of CHOMg molecules within organic chemical evolution as they may have contributed in carbon sequestration within geochemical timescales.

## 5. Conclusions

Astrochemistry is extremely rich in complex organic molecules, as observed, e.g., in the interstellar medium or via meteoritic analyses. The detection of these complex and diverse organic and metalorganic chemical spaces requires high quality chemical analytics, in terms of resolution, accuracy, precision and sensitivity. The high molecular diversity (>15,000 molecular formulas) of extraterrestrial organic matter that has been found in meteorites represents a chemodiversity hotspot. To address questions like “How did this complex organic material form and what role has this material played in the origin of life on Earth?”, ultrahigh-resolving chemical analytics like FT-ICR mass spectrometry need to be applied. Such kinds of analyses produce high-dimensional and complex chemical datasets. To access information from these measurements, sophisticated data analytical methods (e.g., molecular networks) help to expand our knowledge in astrochemistry toward higher molecular masses and complex molecular structures. Increased knowledge of these building blocks of life might help in extending our fundamental understanding on the emergence of life and its chemical processes.

## Figures and Tables

**Table 1:** Molecules in the interstellar medium or circumstellar shells (as of October 2017) [2].

Atom Number	Molecules
2 atoms	H${}_{2}$; AlF; AlCl; C${}_{2}$ **; CH; CH${}^{+}$; CN; CO; CO${}^{+}$; CP; SiC; HCl; KCl; NH; NO; NS;
	NaCl; OH; PN; SO; SO${}^{+}$; SiN; SiO; SiS; CS; HF; HD; FeO?; O${}_{2}$; CF${}^{+}$; SiH?; PO;
	AlO; OH${}^{+}$; CN${}^{-}$; SH${}^{+}$; SH; HCl${}^{+}$; TiO; ArH${}^{+}$; N${}_{2}$; NO${}^{+}$ ?
3 atoms	C${}_{3}$ *; C${}_{2}$H; C${}_{2}$O; C${}_{2}$S; CH${}_{2}$; HCN; HCO; HCO${}^{+}$; HCS${}^{+}$; HOC${}^{+}$; H${}_{2}$O; H${}_{2}$S; HNC;
	HNO; MgCN; MgNC; N${}_{2}$H${}^{+}$; N${}_{2}$O; NaCN; OCS; SO${}_{2}$; c-SiC${}_{2}$; CO${}_{2}$ *; NH${}_{2}$; H${}_{3}$${}^{+}$ (*);
	SiCN; AlNC; SiNC; HCP; CCP; AlOH; H2O${}^{+}$; H${}_{2}$Cl+; KCN; FeCN; HO${}_{2}$; TiO${}_{2}$;
	C${}_{2}$N; Si${}_{2}$C 2015
4 atoms	c-C${}_{3}$H; l-C${}_{3}$H; C${}_{3}$N; C${}_{3}$O; C${}_{3}$S; C${}_{2}$H${}_{2}$ *; NH${}_{3}$; HCCN; HCNH${}^{+}$; HNCO; HNCS;
	HOCO${}^{+}$; H${}_{2}$CO; H${}_{2}$CN; H${}_{2}$CS; H${}_{3}$O${}^{+}$; c-SiC${}_{3}$; CH${}_{3}$ *; C${}_{3}$N${}^{-}$; PH${}_{3}$; HCNO; HOCN;
	HSCN; H${}_{2}$O${}_{2}$; C${}_{3}$H${}^{+}$; HMgNC; HCCO 2015
5 atoms	C${}_{5}$ *; C${}_{4}$H; C${}_{4}$Si; l-C${}_{3}$H${}_{2}$; c-C${}_{3}$H${}_{2}$; H${}_{2}$CCN; CH${}_{4}$ *; HC${}_{3}$N; HC${}_{2}$NC; HCOOH;
	H${}_{2}$CNH; H${}_{2}$C${}_{2}$O; H${}_{2}$NCN; HNC${}_{3}$; SiH${}_{4}$ *; H${}_{2}$COH${}^{+}$; C${}_{4}$H${}^{-}$; HC(O)CN; HNCNH;
	CH${}_{3}$O; NH${}_{4}$${}^{+}$; H${}_{2}$NCO${}^{+}$ (?); NCCNH${}^{+}$ 2015; CH${}_{3}$Cl 2017
6 atoms	C${}_{5}$H; l-H${}_{2}$C${}_{4}$; C${}_{2}$H${}_{4}$ *; CH${}_{3}$CN; CH${}_{3}$NC; CH${}_{3}$OH; CH${}_{3}$SH; HC${}_{3}$NH${}^{+}$; HC${}_{2}$CHO;
	NH${}_{2}$CHO; C${}_{5}$N; l-HC${}_{4}$H *; l-HC${}_{4}$N; c-H${}_{2}$C${}_{3}$O; H${}_{2}$CCNH (?); C${}_{5}$N${}^{-}$; HNCHCN;
	SiH${}_{3}$CN 2017
7 atoms	C${}_{6}$H; CH${}_{2}$CHCN; CH${}_{3}$C2H; HC${}_{5}$N; CH${}_{3}$CHO; CH${}_{3}$NH${}_{2}$; c-C${}_{2}$H${}_{4}$O; H${}_{2}$CCHOH;
	C${}_{6}$H${}^{-}$; CH${}_{3}$NCO 2015; HC${}_{5}$O 2017
8 atoms	CH${}_{3}$C${}_{3}$N; HC(O)OCH${}_{3}$; CH${}_{3}$COOH; C${}_{7}$H; C${}_{6}$H${}_{2}$; CH${}_{2}$OHCHO; l-HC${}_{6}$H *;
	CH${}_{2}$CHCHO (?); CH${}_{2}$CCHCN; H${}_{2}$NCH${}_{2}$CN; CH${}_{3}$CHNH; CH${}_{3}$SiH${}_{3}$ 2017
9 atoms	CH${}_{3}$C${}_{4}$H; CH${}_{3}$CH${}_{2}$CN; (CH${}_{3}$)${}_{2}$O; CH${}_{3}$CH${}_{2}$OH; HC${}_{7}$N; C${}_{8}$H; CH${}_{3}$C(O)NH${}_{2}$;
	C${}_{8}$H${}^{-}$; C${}_{3}$H${}_{6}$; CH${}_{3}$CH${}_{2}$SH (?); CH${}_{3}$NHCHO ? 2017
10 atoms	CH${}_{3}$C${}_{5}$N; (CH${}_{3}$)${}_{2}$CO; (CH${}_{2}$OH)${}_{2}$; CH${}_{3}$CH${}_{2}$CHO; CH${}_{3}$CHCH${}_{2}$O 2016
11 atoms	HC${}_{9}$N; CH${}_{3}$C${}_{6}$H; C${}_{2}$H${}_{5}$OCHO; CH${}_{3}$OC(O)CH${}_{3}$
12 atoms	c-C${}_{6}$H${}_{6}$ *; n-C${}_{3}$H${}_{7}$CN; i-C${}_{3}$H${}_{7}$CN; C${}_{2}$H${}_{5}$OCH${}_{3}$ ?
>12 atoms	HC${}_{11}$N ?; C${}_{60}$ *; C${}_{70}$ *; C${}_{60}$${}^{+}$ *

* Indicates molecules that have been detected by their rotation-vibration spectrum; ** those detected by electronic spectroscopy only, tentative detections, which have a reasonable chance to be correct, are indicated by “?”. Depicted dates represent the year most relevant to the detection (including isotopic species or vibrationally-excited states) given for recent results: the past two to three years.
